# Magnetic resonance imaging-based radiomics signature for preoperative prediction of Ki67 expression in bladder cancer

**DOI:** 10.1186/s40644-021-00433-3

**Published:** 2021-12-04

**Authors:** Zongtai Zheng, Zhuoran Gu, Feijia Xu, Niraj Maskey, Yanyan He, Yang Yan, Tianyuan Xu, Shenghua Liu, Xudong Yao

**Affiliations:** 1 Department of Urology, Shanghai Tenth People’s Hospital, School of Medicine, Tongji University, Yan Chang Zhong Road 301, Shanghai, 200072 China; 2 Institute of Urinary Oncology, School of Medicine, Tongji University, Yan Chang Zhong Road 301, Shanghai, 200072 China; 3 Department of Radiology, Shanghai Tenth People’s Hospital, School of Medicine, Tongji University, Shanghai, China; 4 Department of Pathology, Shanghai Tenth People’s Hospital, School of Medicine, Tongji University, Shanghai, China

**Keywords:** Bladder cancer, Magnetic resonance imaging, Ki67, Radiomics

## Abstract

**Purpose:**

The Ki67 expression is associated with the advanced clinicopathological features and poor prognosis in bladder cancer (BCa). We aimed to develop and validate magnetic resonance imaging (MRI)-based radiomics signatures to preoperatively predict the Ki67 expression status in BCa.

**Methods and materials:**

We retrospectively collected 179 BCa patients with Ki67 expression and preoperative MRI. Radiomics features were extracted from T2-weighted (T2WI) and dynamic contrast-enhancement (DCE) images. The synthetic minority over-sampling technique (SMOTE) was used to balance the minority group (low Ki67 expression group) in the training set. Minimum redundancy maximum relevance was used to identify the best features associated with Ki67 expression. Support vector machine and Least Absolute Shrinkage and Selection Operator algorithms (LASSO) were used to construct radiomics signatures in training and SMOTE-training sets, and diagnostic performance was assessed by the area under the curve (AUC) and accuracy. The decision curve analyses (DCA) and calibration curve and were used to investigate the clinical usefulness and calibration of radiomics signatures, respectively. The Kaplan-Meier test was performed to investigate the prognostic value of radiomics-predicted Ki67 expression status.

**Results:**

1218 radiomics features were extracted from T2WI and DCE images, respectively. The SMOTE-LASSO model based on nine features achieved the best predictive performance in the SMOTE-training (AUC, 0.859; accuracy, 80.3%) and validation sets (AUC, 0.819; accuracy, 81.5%) with a good calibration performance and clinical usefulness. Immunohistochemistry-based high Ki67 expression and radiomics-predicted high Ki67 expression based on the SMOTE-LASSO model were significantly associated with poor disease-free survival in training and validation sets (all *P* < 0.05).

**Conclusions:**

The SMOTE-LASSO model could predict the Ki67 expression status and was associated with survival outcomes of the BCa patients, thereby may aid in clinical decision-making.

**Supplementary Information:**

The online version contains supplementary material available at 10.1186/s40644-021-00433-3.

## Introduction

Bladder cancer (BCa) is the 10th most prevalent cancer with high risk of malignant progression, metastasis and recurrence [1]. BCa can be classified into non-muscle-invasive bladder cancer (NMIBC) and muscle-invasive bladder cancer (MIBC) based on the muscle invasion status. At initial diagnosis, approximately 75% of BCa patients represent NMIBC (Ta, Tis, T1) while the remaining 25% accounts for MIBC (stage from T2 to T4) [2].

Ki67 nucleoprotein, an indicator of cell growth fraction and a marker associated with proliferative activity of cell, presents the G1 stage (prophase of DNA synthesis) to mitosis of the cell cycle [3]. Previous studies have demonstrated that high Ki67 expression is associated with higher T stage, higher tumor grade, lymph nodes invasion, lymphovascular invasion, and poorer prognosis in BCa [4–8]. More interestingly, a meta-analysis study has reported that high expression of Ki67 was a risk factor for progression-free survival in NMIBC patients treated with transurethral resection and Bacillus Calmette-Guérin intravesical immunotherapy [9]. Therefore, Ki67 expression is not only a useful indicator of tumor characteristics and prognosis, but also may be a reference tool for treatment decision making. In BCa, the Ki67 expression can only be postoperatively detected by immunohistochemistry (IHC) using samples from either radical cystectomy or cystoscopic biopsy. However, due to heterogeneity in the BCa samples and relatively small sample size, the examination of Ki67 expression using cystoscopic biopsy may not represent entire BCa, which limits its application. Therefore, a noninvasive and accurate tool is needed to preoperatively predict the Ki67 expression in BCa patients more comprehensively and accurately.

With the development of imaging techniques and postprocessing analysis, Magnetic resonance imaging (MRI) is becoming a routine and useful non-invasive tool for preoperative tumor diagnosis and clinical staging in BCa. One promising method to optimize radiological assessment for Ki67 expression prediction is the application of radiomics that has rapidly developed in the field of medical imaging analysis in recent years and has been widely utilized for the prediction of the biological behavior in various tumors [10–12]. Compared with imaging characteristics generated by subjective evaluation, radiomics is more objective and can extract high-dimensional imaging features that could not be detected by human eyes and might be correlated with the intratumor heterogeneity [13]. In addition, it is also a preoperative and non-invasive method for the evaluation of tumor heterogeneity. Previous studies have constructed CT/MRI based radiomics signature for biological behaviors prediction in BCa, including muscle-invasive status, lymph node metastasis, tumor stage, prognosis and therapeutic response [14–18], which suggests that radiomics features may potentially predict the expression of Ki67 in BCa for the sake of positive relationship between Ki67 expression and malignant progression [4–6, 9].

In BCa, T2-weighted (T2WI) is usually used to evaluate location, tumor size, morphology, growth pattern and the degree of interruption of the hypointense muscle, and dynamic contrast-enhancement (DCE) is usually used to evaluate the extension of the early enhancing lesion into the non-early enhancing muscle [19]. Previous studies have used the T2WI- and DCE-based radiomics features to preoperatively predict the muscle-invasive status and pathological grade in BCa [20, 21], suggesting that the T2WI- and DCE-based radiomics features can indicate the biological behavior and heterogeneity on the onset of tumor and may facilitate the application of T2WI- and DCE-based radiomics features for Ki67 expression prediction in BCa.

To the best of our knowledge, no radiomics signatures have been constructed for predicting the Ki67 expression in BCa. In this study, we adopted the radiomics to extract high-throughput features from T2WI and DCE images and used Support vector machine (SVM) and Least Absolute Shrinkage and Selection Operator (LASSO) algorithms to construct radiomics signatures to preoperatively predict the Ki67 expression status and investigate their prognostic value in BCa.

## Materials and methods

### Patients

In this retrospective cohort study, BCa patients who were diagnosed by pathology between August 2014 and April 2020 were retrospectively collected from our center. The inclusion criteria included the following: (1) BCa patients who underwent radical cystectomy or transurethral resection; (2) BCa was diagnosed with histopathology and IHC; (3) MRI examinations were performed < 20 days ahead of surgery; (4) No missing prognostic information and Ki67 expression. The exclusion criteria included the following: (1) Poor-quality MRI images; (2) Chemotherapy or radiotherapy were performed before multiparametric pelvic MRI; (3) Lesions for which it was difficult to define the boundaries. Before the surgical resection, the MRI-determined clinical factors, including hydronephrosis, tumor size, number of tumors, the Vesical Imaging-Reporting and Data System (VI-RADS) score and clinical T stage, were evaluated by two radiologists. VI-RADS is becoming an imaging protocol and reporting criterion for bladder MRI and provides five-point scores that predict the possibility of muscle invasiveness by BCa [22]. The protocols of MRI examination were available in our previous methods [23]. In this study, Digital Imaging and Communications in Medicine images (DICOM) were retrieved for the radiomics analysis. The disease-free survival (DFS) of patients was the time when a patient suffers from the first recurrence, or first progression, including metastasis or death after the initiation of surgery. Tumor recurrence or tumor progression was diagnosed based on patients’ symptoms and medical images. Follow-up was performed every 3–6 months after surgery via telephone call or hospital visit to obtain the DFS of patients.

### Tumor segmentation and feature extraction

One radiologist (F Xu, with over 5-year experience in bladder MRI reading) segmented the region of interest (ROI). For each BCa patient, the boundaries of tumor were drew on each slice on the DCE images and T2WI images using ITK-SNAP software (version 3.6.0; http://itk-snap.org). The areas of vessels or necrosis were excluded. When multiple tumors existed for a patient, the maximal lesion was segmented for features extraction [24, 25] Volumes of interest (VOI) was constructed by stacking up the ROIs of each patient. After 30 days, the same radiologist and another radiologist (T Xu, with over 10-year experience in bladder MRI reading) repeatedly segmented the VOIs of 40 randomly selected BCa patients to evaluate the intra- and inter-observer agreement on feature extraction. In this process, two radiologists were blind to the prognostic information and Ki67 expression.

Before radiomics features extraction, all the DICOMs were subjected to image normalization and resampled to the same resolution (1 mm × 1 mm × 1 mm) to avoid data heterogeneity. Four classes of radiomics features (including shape and size, first-order features, textural features and wavelet features) were extracted from segmented tumors using the PyRadiomics platform (http://www.radiomics.io/pyradiomics.html). Totally 2436 radiomics features were extracted from the axial T2WI and delay phase of DCE images. Radiomics features of all patients were standardized by using the Z-score [(x – μ)/σ]. In this formula, x is the radiomics feature value, μ is the mean of the feature values and σ is the corresponding standard deviation. μ and σ were calculated based on the training set.

### Feature selection

The intra- and interclass correlation coefficients (ICCs) were used to evaluate the intra- and inter-observer agreement on feature extraction. Features with ICC > 0.75 were selected for the minimum redundancy maximum relevance (mRMR). mRMR is a supervised feature selection algorithm which calculates the mutual information (MI) between a target variable and features. It ranks features via maximizing MI with respect to the target variable and then minimizes the average MI for features with higher rankings [26].

### Assessment of Ki67

**Fig. 1:**
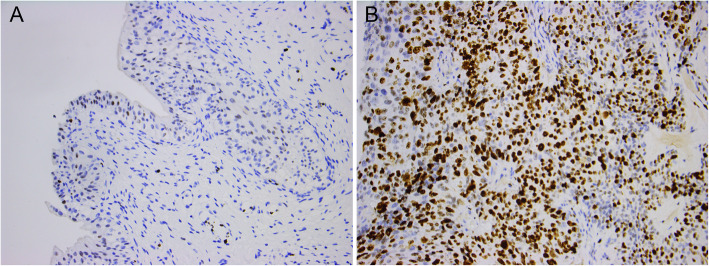
Representative immunostaining (original magnification, × 200) for Ki67 in bladder cancer. **a** Low Ki67 expression. **b** High Ki67 expression

After surgical resection, IHC was performed on BCa samples for assessment of the Ki67 within a week. Mouse anti-human monoclonal primary antibodies against Ki67 (Bio-Rad Cat# MCA289, RRID:AB_321740) was utilized to detect Ki67 expression according to the manufacturer’s protocol. Immunoreactivity for Ki67 was scored according to the Ki67 positive cells among randomly selected 1000 cells in each section by two independent pathologists who were blind to the prognostic information and clinical data. According to previous studies [4, 6, 7], BCa patients were divided into two groups: high Ki67 expression group (>15% cells stained) and low Ki67 expression group (≤15% cells stained) (Fig. 1).

### Data balancing and radiomics signatures construction

BCa patients were randomly allocated into training set and validation set based on a 7:3 ratio. The ratio of low Ki67 expression patients to high Ki67 expression patients was 1:3.81 (26 low Ki67 expression patients and 99 high Ki67 expression patients) in the training set, revealing a sample imbalance. The synthetic minority over-sampling technique (SMOTE) algorithm was used to balance the minority class in the training set [27], so that the two classes of BCa patients were 1:1 (99 low Ki67 expression patients and 99 high Ki67 expression patients) in the SMOTE-training set. We developed four radiomics signatures, including SVM and LASSO models in the training set and SMOTE-SVM and SMOTE-LASSO models in the SMOTE-training set. These radiomics signatures were all validated in the validation set (15 low Ki67 expression patients and 39 high Ki67 expression patients).

The nonlinear SVM-based recursive feature elimination (SVM-RFE) algorithm was applied to select the optimal number of features and the most relevant features for SVM model development via 10-fold cross-validation [28]. The kernel parameters of SVM model were computed inside the folds with a standard grid search procedure and the prediction of Ki67 expression was automatically determined by the SVM model. The SVM model generated an internal score called decision value that was used as the radiomics score of SVM. The LASSO algorithm was conducted to omit features that minimally related to the target variable and obtain features with non-zero coefficients via 10-fold cross-validation [29]. The radiomics score of each patient was calculated by summing the selected radiomics features weighted by their coefficients. To classifying BCa patients into radiomics-predicted low and high Ki67 expression groups, the optimal cutoff value of radiomics score in LASSO model was calculated with the highest Youden index.

The performance of radiomics signatures was evaluated by accuracy, sensitivity, specificity, negative-predictive value (NPV), and positive-predictive value (PPV) based on the Youden index. The area under the receiver operator characteristic (ROC) curve (AUC) was also calculated for radiomics signatures. Decision curve analysis (DCA) and calibration curves were conducted to investigate the clinical usefulness and calibration of the radiomics signature, respectively.

### Statistical analysis

Statistical analysis was conducted with SPSS 23.0 (SPSS, Armonk, NY, USA) and R statistical software (version 3.6.1 R, https://www.r-project.org/). The R packages used in this study were showed in Supplemental Table 1. The clinical characteristics between the training and validation sets were compared applying the Student’s t-test, the Chi-square test, or the Mann-Whitney U test, as appropriate. The Kaplan-Meier and log-rank tests were performed between two groups, defined by IHC-based Ki67 expression status and radiomics-predicted Ki67 expression status, respectively. All tests were 2-tailed, and *P* value< 0.05 was regarded as statistically significant.

## Results

### Patient population

**Fig. 2:**
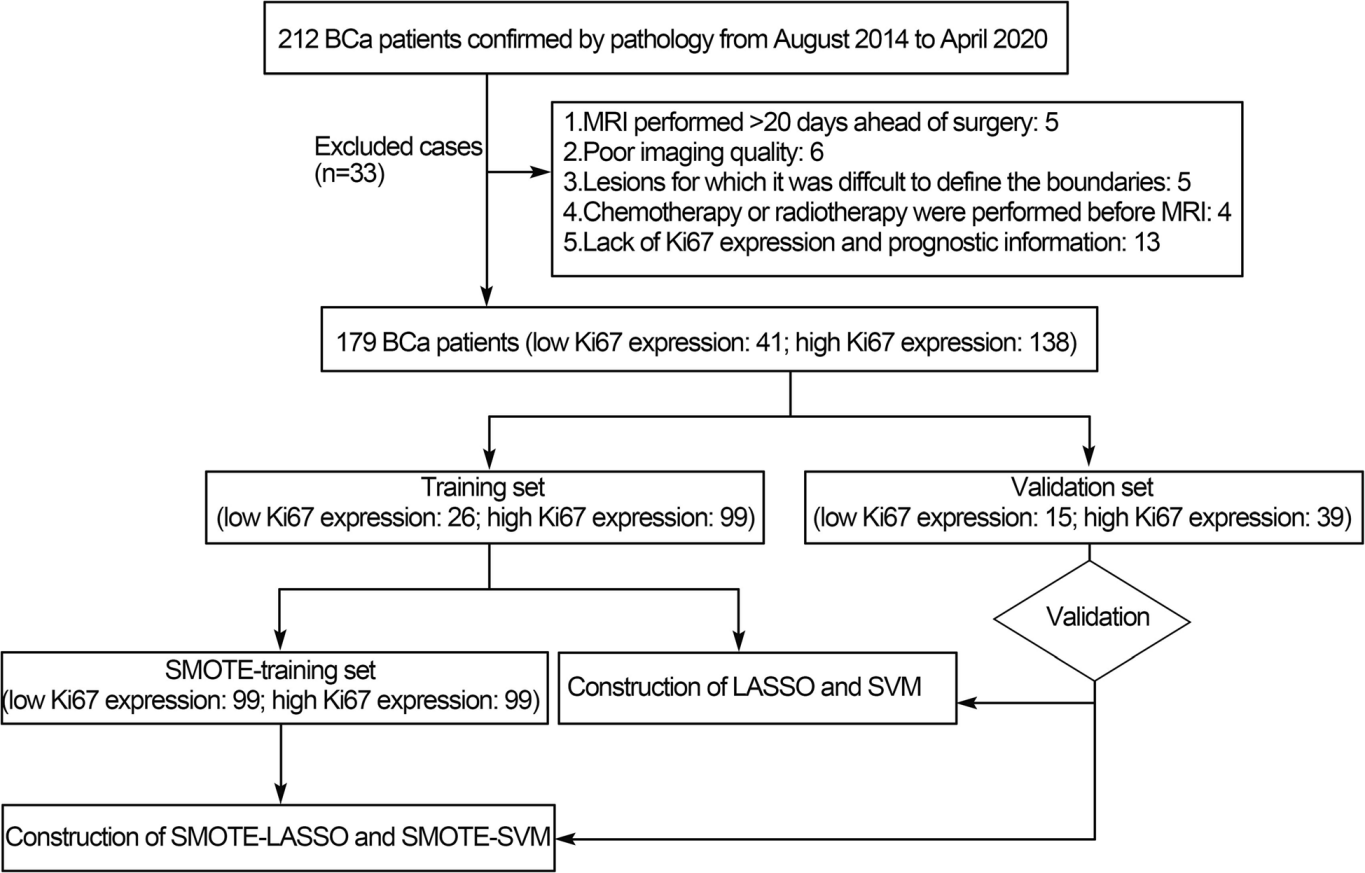
The study flow chart of the study. BCa: bladder cancer; MRI: magnetic resonance imaging; SMOTE: synthetic minority over-sampling technique

A total of 179 BCa patients [147 male, 32 female; mean age = 67.8 years ±12.1 (standard deviation), range 20–93 years; 120 NMIBC, 59 MIBC] were collected after excluding 33 patients who did not meet the inclusion criteria. Of the 179 BCa patients, 41 were low Ki67 expression, and 138 were high Ki67 expression. The specific flow chart was presented in Fig. 2. There were 125 (high Ki67 expression: 99 patients; low Ki67 expression: 26 patients) and 54 (high Ki67 expression: 39 patients; low Ki67 expression: 15 patients) patients in training and validation sets, respectively. There were no significant differences in age, sex, MRI-determined clinical T stage, tumor number, tumor size, hydronephrosis and VI-RADS score between two data sets (Table 1).

**Table 1:** Clinicopathological characteristics of patients

Characteristic	Number of Patients (%)		*P* value
	Training Set (*n* = 125)	Validation Set (*n* = 54)	
Sex			
Men	106 (84.8)	41 (75.9)	0.155^b^
Women	19 (15.2)	13 (24.1)	
Age (years)			
< 65	42 (33.6)	24 (44.4)	0.681^b^
≥ 65	83 (66.4)	30 (55.6)	
Hydronephrosis^a^			
Yes	20 (16.0)	12 (22.2)	0.319^b^
No	105 (84.0)	42 (77.8)	
Tumor size^a^ (cm)			
< 3	82 (65.6)	35 (64.8)	0.919^b^
≥ 3	43 (34.4)	19 (35.2)	
Number of tumors^a^			
Single	88 (70.4)	36 (66.7)	0.619^b^
Multiple	37 (29.6)	18 (33.3)	
VI-RADS score^a^			
1	15 (12.0)	7 (13.0)	0.824^c^
2	35 (28.0)	12 (22.2)	
3	35 (28.0)	17 (31.5)	
4	16 (12.8)	9 (16.7)	
5	24 (19.2)	9 (16.7)	
Clinical T stage^a^			
< T2	85 (68.0)	35 (64.8)	0.677^b^
≥ T2	40 (32.0)	19 (35.2)	

VI-RADS, Vesical Imaging-Reporting and Data System.

^a^ MRI-determined information

^b^ Statistical analysis performed using chi-square test.

^c^ Statistical analysis performed using Mann-Whitney U test.

A total of 2436 radiomics features were extracted from axial DCE and T2WI sequences (1218 features per sequence). According to the standard of ICC > 0.75, 1136 features from DCE images and 1166 features from T2WI images were highly robust and chosen for subsequent analyses. The top 10 features were selected by mRMR for SMOTE-based data balancing and radiomics signatures construction. These processes were performed in the training set. After data balancing, the number of patients in SMOTE-training set were 198 (high Ki67 expression: 99 patients; low Ki67 expression: 99 patients).

### Radiomics signatures development

**Fig. 3:**
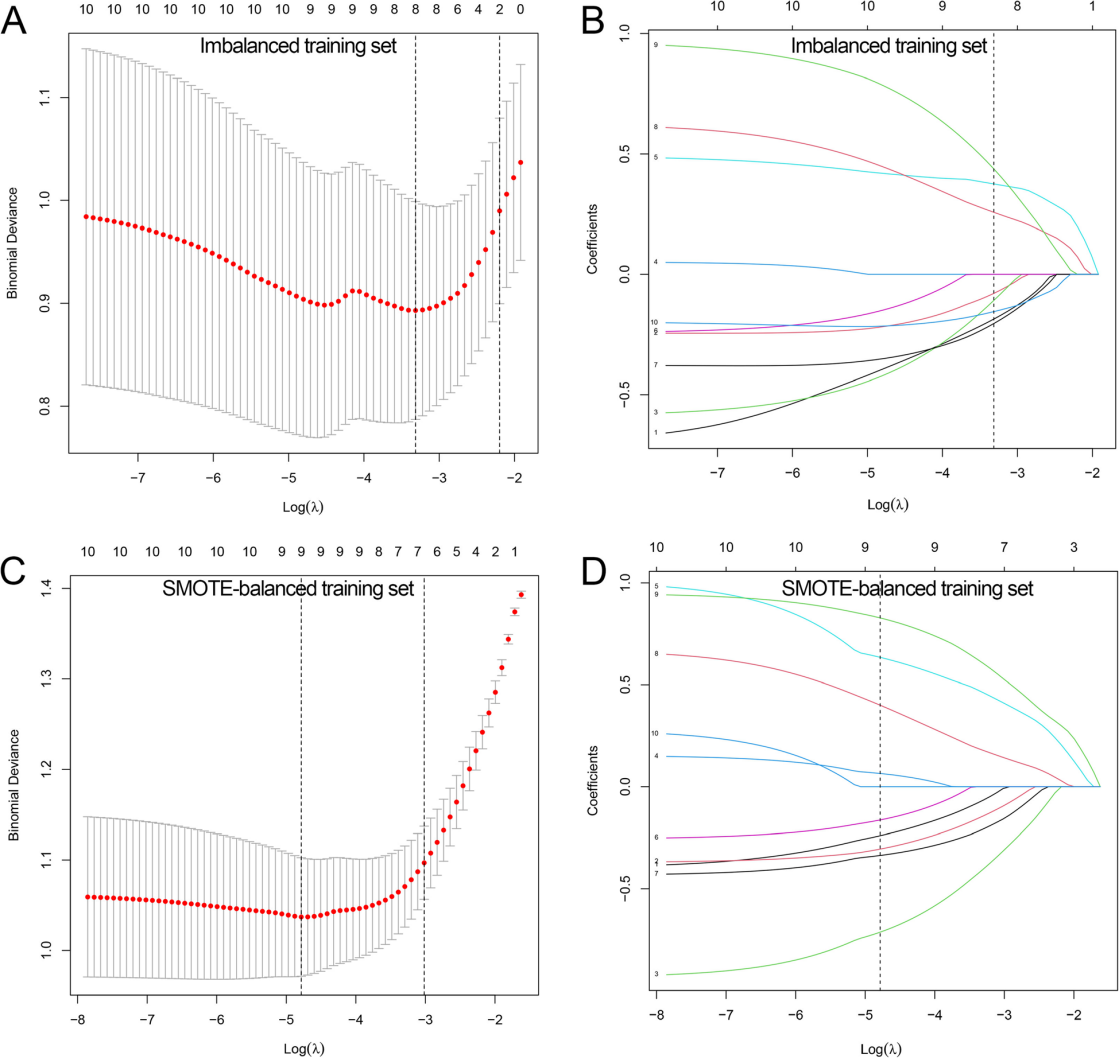
Development of LASSO models. **a** Selecting the optimal number of features based on minimum criteria in the training set. **b** Based on the optimal λ value of 0.036 with log(λ) = − 3.315, eight features were selected. **c** Selecting the optimal number of features based on minimum criteria in the SMOTE-training set. **d** Based on the optimal λ value of 0.008 with log(λ) = − 4.785, nine features were selected. LASSO: least absolute shrinkage and selection operator; SMOTE: synthetic minority over-sampling technique

**Fig. 4:**
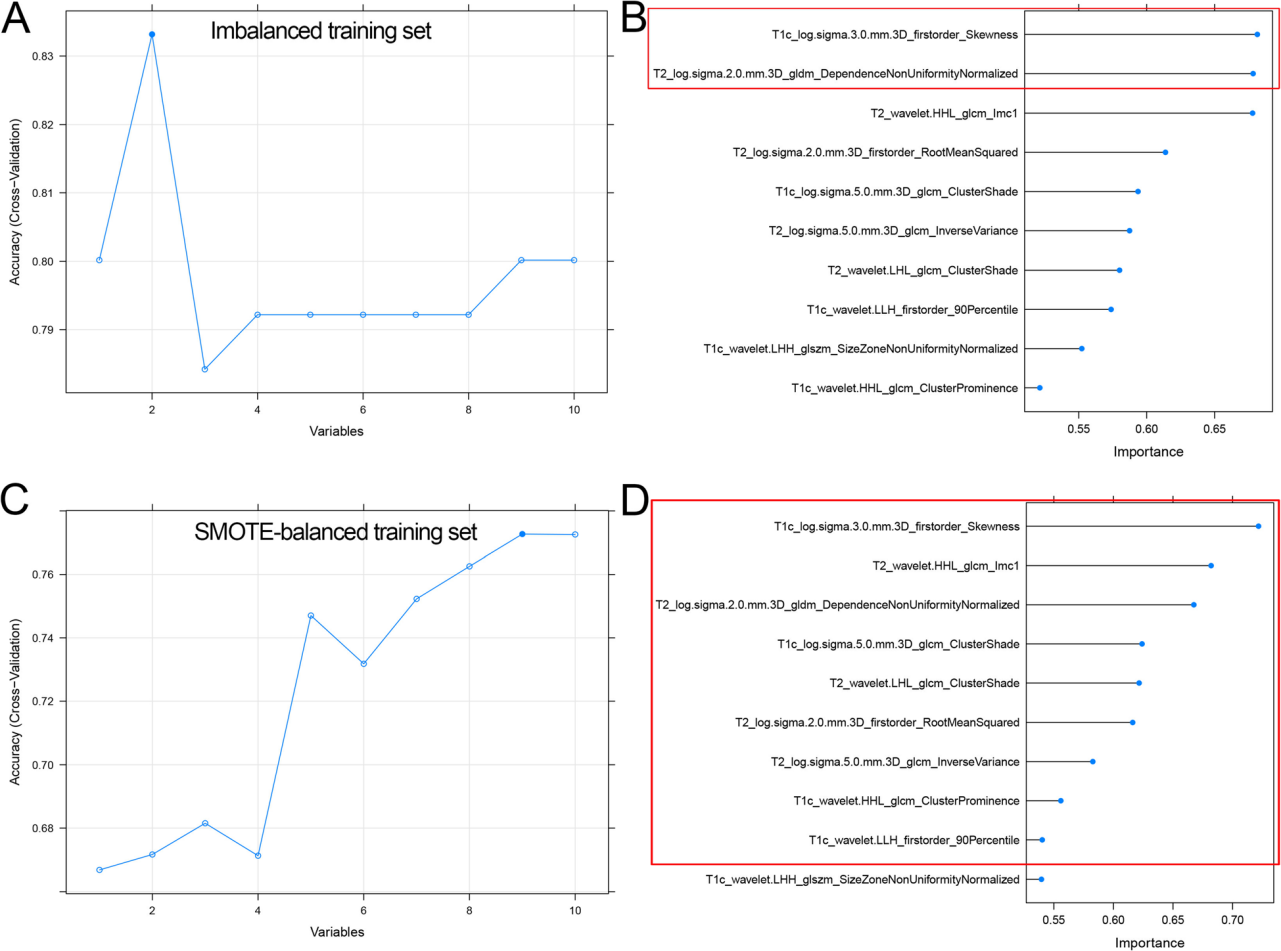
Development of SVM models. **a** Selecting the optimal number of features (two features) using SVM-RFE in the training set. **b** Features were ranked according to the feature importance by SVM-RFE, and the top two features were selected for SVM model construction. **c** Selecting the optimal number of features (nine features) in the SMOTE-training set. **d** The top nine features were selected for SVM model construction. SVM: support vector machine; SVM-RFE: SVM-based recursive feature elimination; SMOTE: synthetic minority over-sampling technique

Eight and nine features with non-zero coefficients were chosen by LASSO algorithm to construct LASSO and SMOTE-LASSO models with the least binominal deviance, respectively (Fig. 3). Through the SVM-RFE algorithm, the top two and nine features were used to develop SVM and SMOTE-SVM models with the highest accuracy, respectively (Fig. 4).

### Performance of radiomics signatures

**Fig. 5:**
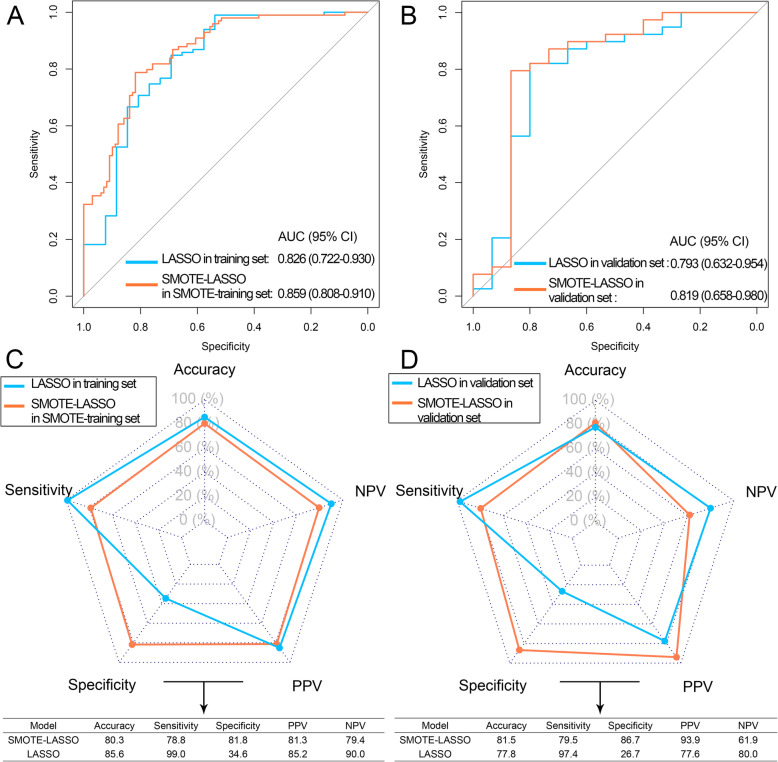
Performance of LASSO models. **a** The ROC curves of LASSO and SMOTE-LASSO models in training and SMOTE-training sets, respectively. **b** The ROC curves of LASSO and SMOTE-LASSO models in validation set. **c** The performance of LASSO and SMOTE-LASSO models in training and SMOTE-training sets, respectively. **d** The performance of LASSO and SMOTE-LASSO models in validation set. ROC: receiver operating curve; AUC: area under the ROC curve; NPV: negative predictive value; PPV: positive predict value; LASSO: least absolute shrinkage and selection operator; SMOTE: synthetic minority over-sampling technique

The AUCs of SMOTE-LASSO model were higher than LASSO model in both training and validation sets (Fig. 5 a, b). Due to the high proportion of high Ki67 expression patients, we observed high sensitivity but obviously low specificity of the LASSO model in training and validation sets (Fig. 5 c, d). In contrast, although the sensitivity of SMOTE-LASSO model in training and validation sets declined, the specificity improved greatly (Fig. 5 c, d). In addition, the accuracy of the SMOTE-LASSO model was improved in the validation set (Fig. 5d).

**Fig. 6:**
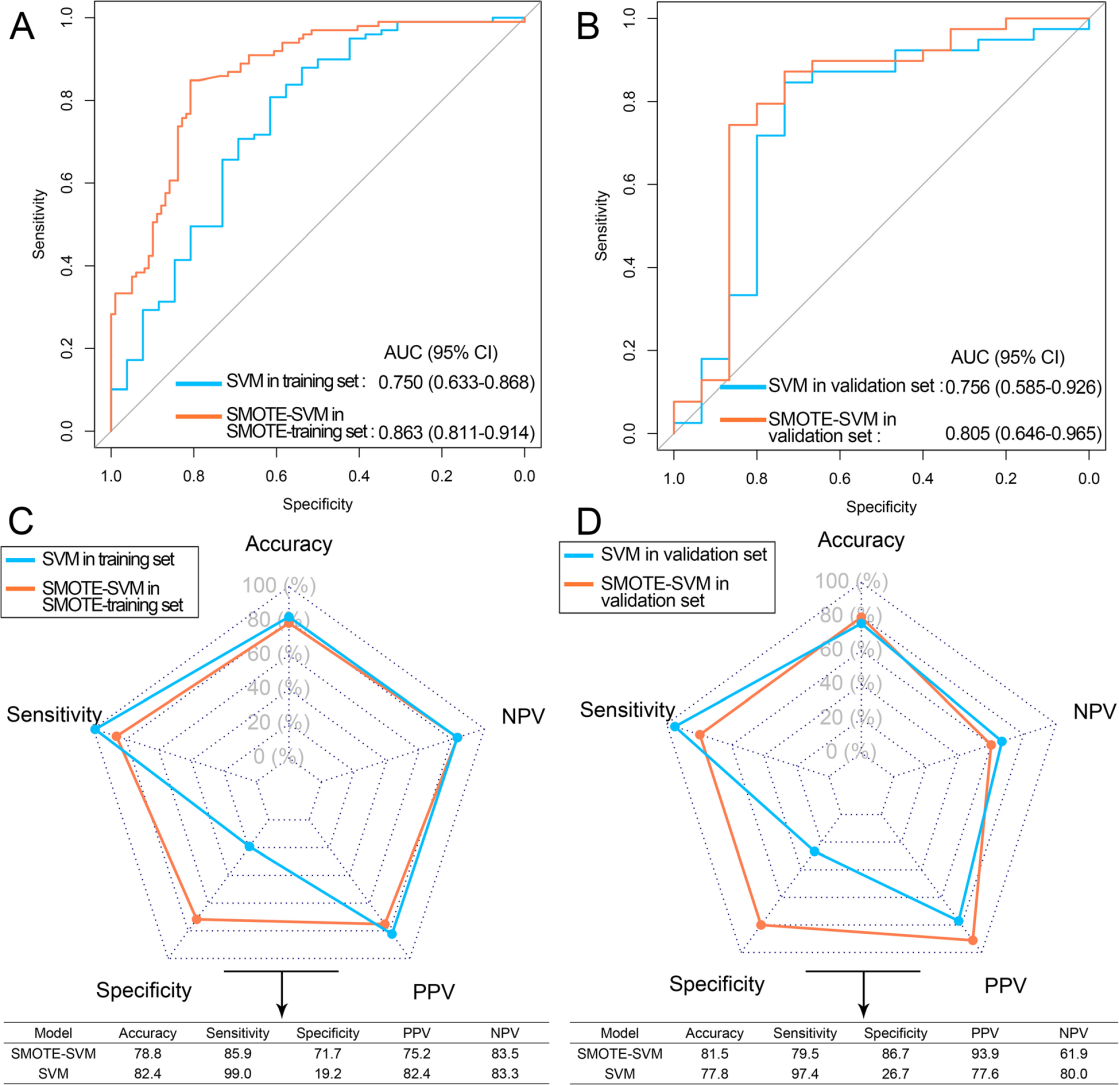
Performance of SVM models. **a** The ROC curves of SVM and SMOTE-SVM models in training and SMOTE-training sets, respectively. **b** The ROC curves of SVM and SMOTE-SVM models in validation set. **c** The performance of SVM and SMOTE-SVM models in training and SMOTE-training sets, respectively. **d** The performance of SVM and SMOTE-SVM models in validation set. ROC: receiver operating curve; AUC: area under the ROC curve; NPV: negative predictive value; PPV: positive predict value; SVM: support vector machine; SMOTE: synthetic minority over-sampling technique

**Fig. 7:**
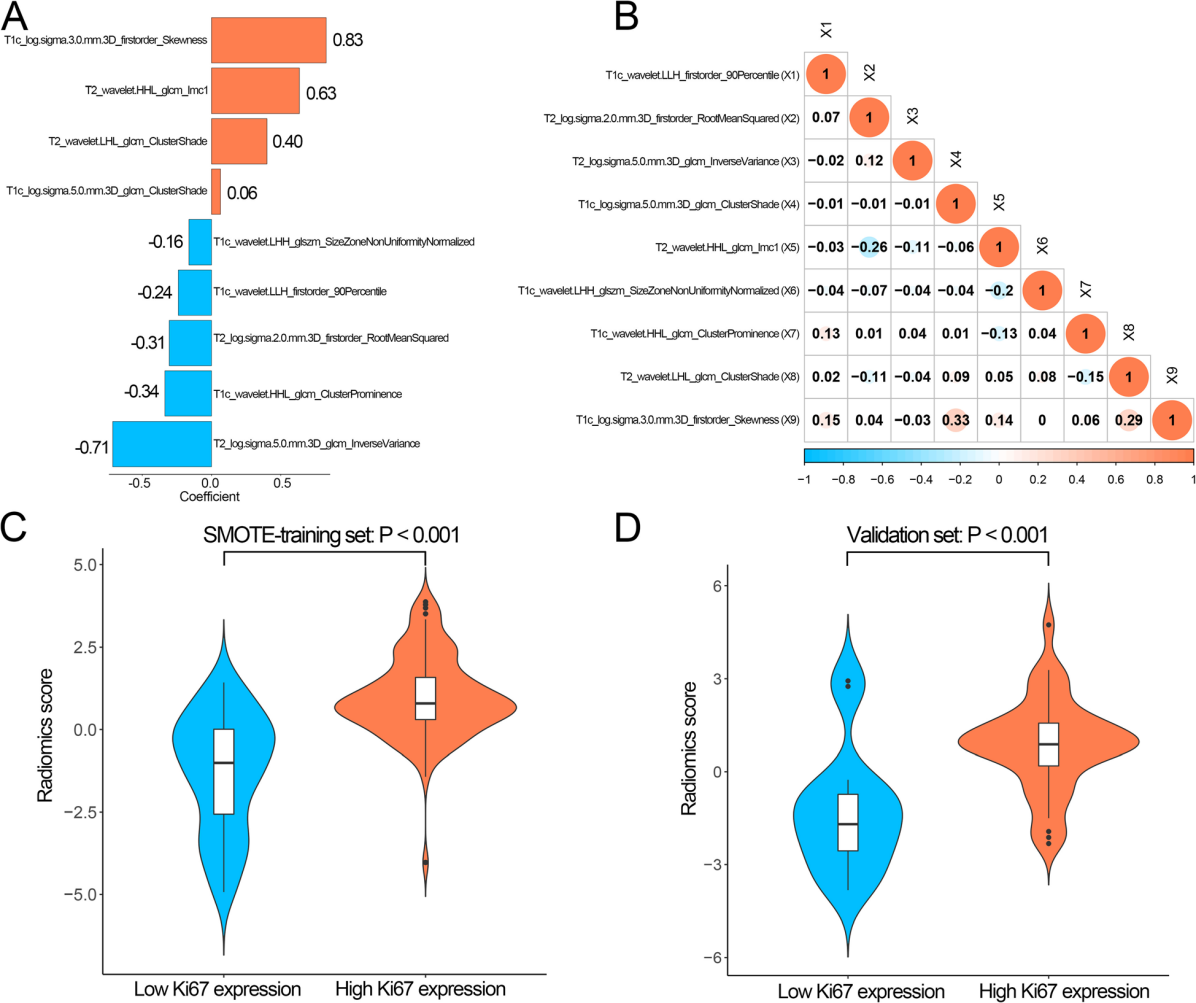
Performance of the radiomics score generated by the SMOTE-LASSO model. **a** The coefficients of nine features in SMOTE-LASSO model. **b** Pairwise Spearman rank correlation among nine features in SMOTE-LASSO model. **c** The violin plot of the radiomics score based on the SMOTE-LASSO model in the SMOTE-training set. **d** The violin plot of the radiomics score based on the SMOTE-LASSO model in the validation set. SVM: support vector machine; SMOTE: synthetic minority over-sampling technique

As for SVM, the AUCs of SMOTE-SVM model were higher than SVM model in both training and validation sets (Fig. 6 a, b), and the specificities of SMOTE-SVM model were improved greatly in training and validation sets (Fig. 6 c, d). However, the accuracy of the SMOTE-SVM model was not improved in the validation set (Fig. 6d).Among these four radiomics signatures, the SMOTE-LASSO achieved the highest AUC in SMOTE-training and validation sets and the highest accuracy in the validation set. In this way, the radiomics signature based on the SMOTE-LASSO model was selected for further analysis. The coefficients of nine features in the SMOTE-LASSO model were showed in Fig. 7a. The nine features were not highly correlated with each other (Fig. 7b, mean absolute Spearman ρ = 0.08).

High Ki67 expression patients had significantly higher radiomics scores based on the SMOTE-LASSO model than low Ki67 expression patients both in the SMOTE-training and validation sets (both *P* < 0.001, Fig. 7 c, d).

**Fig. 8:**
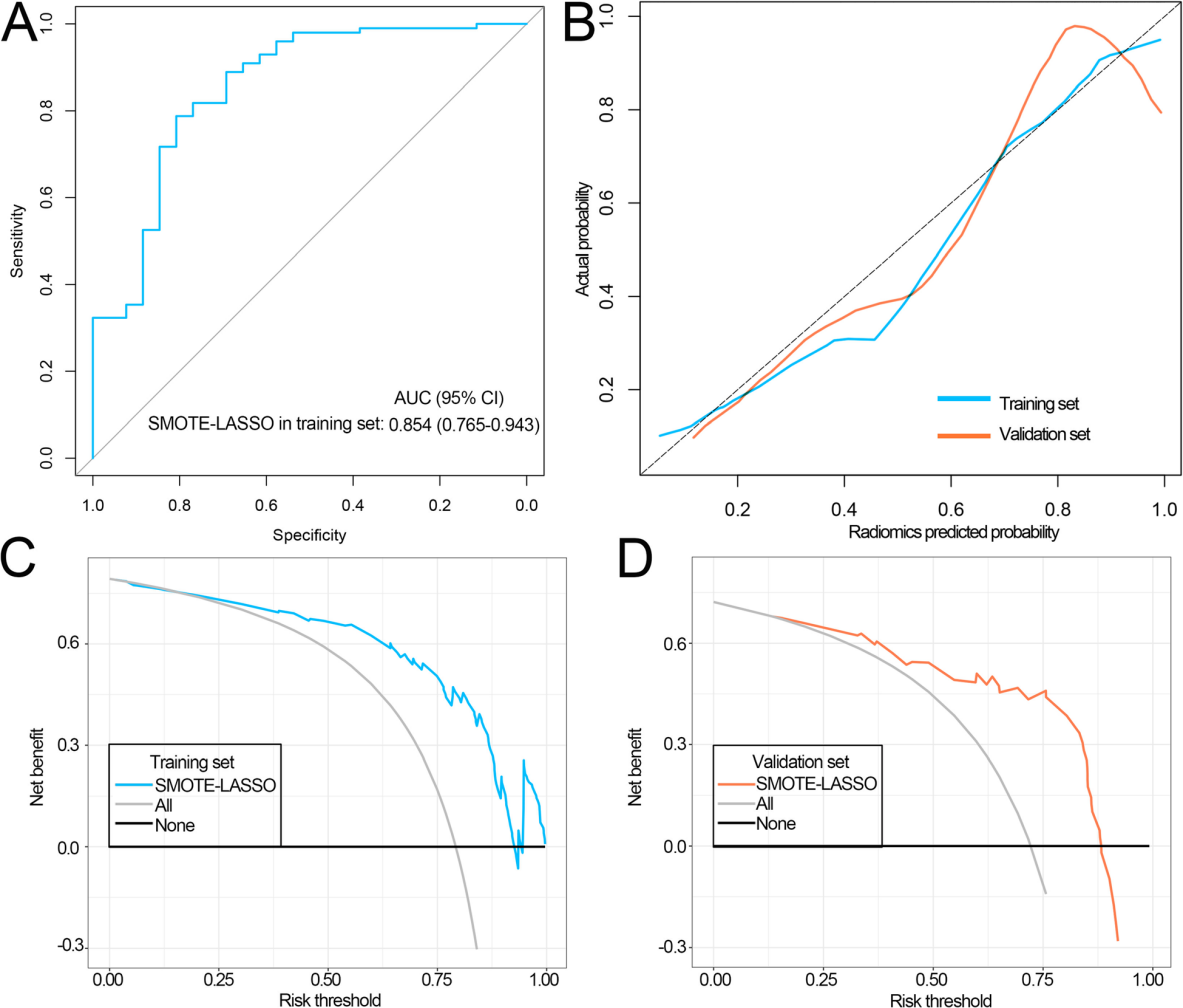
Performance of the radiomics signature based on the SMOTE-LASSO model after omitting the synthetic samples generated by the SMOTE in the SMOTE-training set. **a** The ROC curves of radiomics signature in the SMOTE-training set. **b** Calibration curve of the radiomics signature in SMOTE-training and validation sets. **c-d** DCA for radiomics signature in SMOTE-training **(c)** and validation sets **(d)**. CI: confidence interval; DCA: decision curve analyses; ROC: receiver operating curve; AUC: area under the ROC curve; SMOTE: synthetic minority over-sampling technique

After omitting the synthetic samples in the SMOTE-training set, the AUC of the SMOTE-LASSO model was 0.854 (Fig. 8a, 95% confidence interval: 0.765–0.943). Calibration curves presented a novel agreement between prediction and observation in training and validation sets (Fig. 8b). DCA revealed that the SMOTE-LASSO model based radiomics signature achieved a good clinical net benefit in both data sets (Fig. 8 c, d).

### Relationship between Ki67 expression status and DFS

**Fig. 9:**
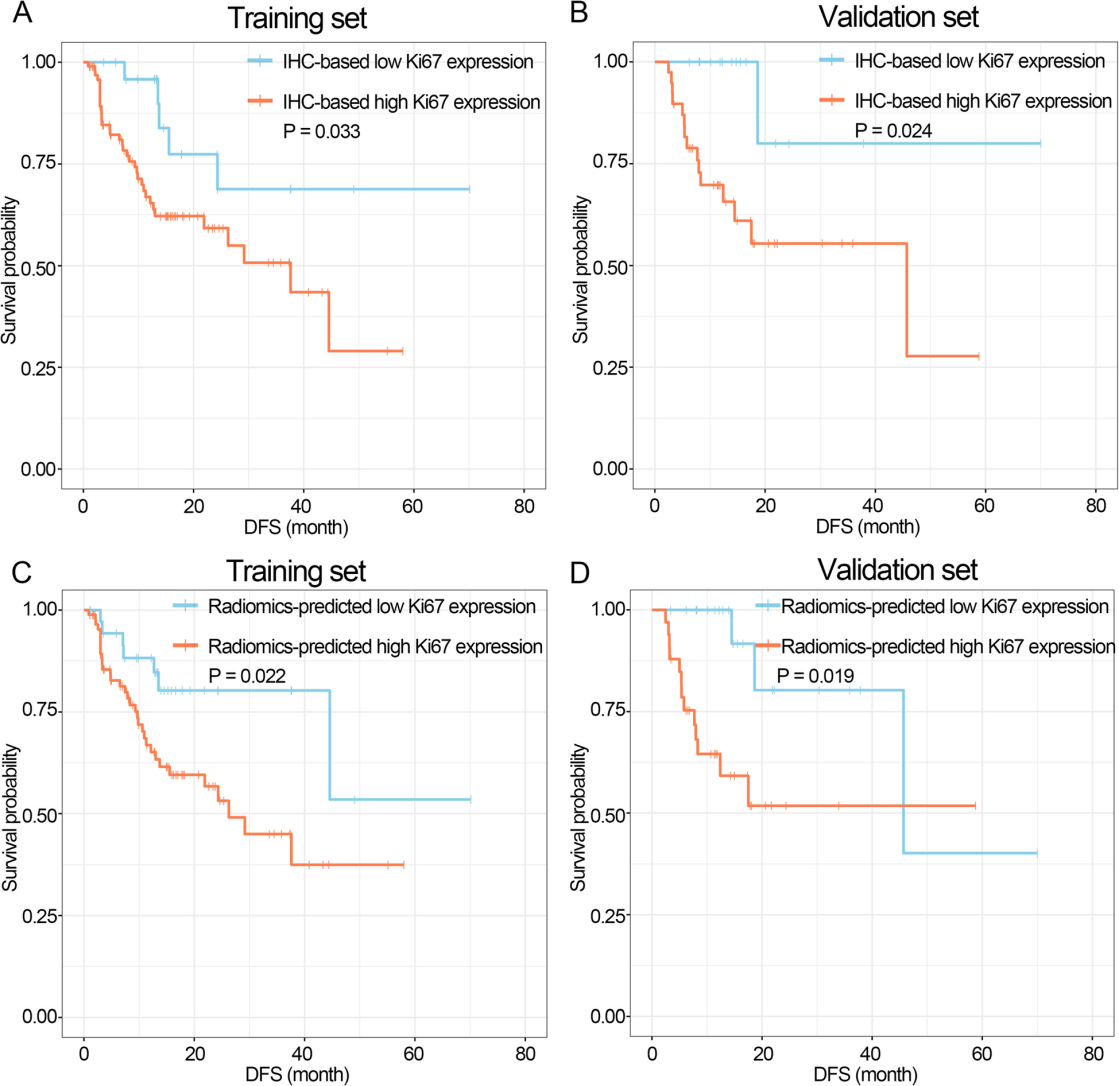
Prognostic value of the IHC-based Ki67 expression status and radiomics-predicted Ki67 expression status based on the SMOTE-LASSO model. **a**, **b** Kaplan-Meier DFS curves for patients grouped by IHC-based Ki67 expression status in training and validation sets, respectively. **c**, **d** Kaplan-Meier DFS curves for patients grouped by radiomics-predicted Ki67 expression status in training and validation sets, respectively.. IHC: immunohistochemistry; DFS: disease-free survival

In this study, the range of follow-up time was 1–70 months, and 56 BCa patients (31.3%) experienced the event of tumor recurrence or tumor progression. BCa patients with IHC-based high Ki67 expression had significantly poor DFS than those with IHC-based low Ki67 expression in training and validation sets (Fig. 9a, b, *P* = 0.033 and 0.024, respectively). We further investigated the association between patients’ survival outcomes and the radiomics-predicted Ki67 expression based on the SMOTE-LASSO model. As a result, BCa patients with radiomics-predicted high Ki67 expression had significantly poor DFS than those with radiomics-predicted low Ki67 expression in training and validation sets (Fig. 9c, d, *P* = 0.022 and 0.019, respectively).

## Discussion

In this retrospective study, we constructed and validated MRI-based radiomics signatures for the preoperative prediction of Ki67 expression status in BCa. Because of the relatively low proportion of low Ki67 expression patients, data were imbalanced between two classes. Without data balancing, the predictive performance of radiomics signatures was inadequate, with obviously low specificity. After data balancing by the SMOTE, the synthesized performance of radiomics signatures was further improved, indicating that data balancing contributes to construct more powerful prediction models. In this study, the SMOTE-LASSO had the optimal performance in the preoperative prediction of Ki67 expression status. Calibration curves presented a novel agreement between the SMOTE-LASSO model based prediction and observation, and DCA revealed that the SMOTE-LASSO model achieved good clinical net benefit. Thus, MRI-based radiomics might assist in preoperatively predicting the Ki67 expression in BCa.

Radiomics is an emerging imaging technique which can extract high-throughput imaging features from medical images and is frequently applied for the prediction of the biological behavior in various tumors [10–12].

The application of radiomics features for the prediction of Ki67 expression status has been reported in gastrointestinal stromal tumor [10], breast cancer [30], thyroid cancer [31], lung cancer [32], liver cancer [33] and glioma [34]. These studies present the value of radiomics in biological behaviors prediction, which may also be a potential method for the prediction of Ki67 expression in BCa on MRI. However, no study has focused on the radiomics features for the prediction of Ki67 expression in BCa. To the best of our knowledge, this study is the first to construct an MRI-based radiomics signature for preoperative Ki67 expression prediction in BCa.

Compared with CT, MRI provides various functional parameters, orientations and angles to comprehensively evaluate the tumor [35]. Most of previous studies also focused on MRI-based radiomics features to predict the Ki-67 expression status in various tumors, including liver cancer [33], thyroid cancer [31], glioma [34] and breast cancer [30, 36], which suggests that MRI-based radiomics features have the potential to predict the Ki67 expression status in BCa. Specifically, T2WI can better evaluate the tumor size and tumor morphology, and the T2WI-based radiomics features have been selected to predict the Ki67 expression in glioma [34] and thyroid cancer [31]. DCE has an advantage of reflecting the tumor microvessel permeability. It is reported that a slight submucosal linear enhancement is associated with nonmuscle invasiveness condition in BCa [37], and the DCE-based radiomics features have been successfully used for the prediction of Ki67 expression in liver cancer [33], breast cancer [30, 36] and thyroid cancer [31], which may facilitate the application of DCE-based features for Ki67 expression prediction in BCa. In this study, the numbers of T2WI-based and DCE-based radiomics features in the nine-feature-based SMOTE-LASSO model were four and five, respectively, revealing that the T2WI-based and DCE-based radiomics features are equally important in Ki67 expression prediction in BCa. In addition, five of nine radiomics features in the SMOTE-LASSO model were wavelet filtered features, revealing that the wavelet transform filter is able to show tumor biology on multiple scales [38]. Wavelet transform filter creates eight decompositions per level in each of the three dimensions and offers high-dimensional radiomics features that remain unnoticed by the human eye. Compared with subjective evaluation by radiologists features or low- dimensional radiomics, wavelet filtered features could provide more information related to biological behavior and heterogeneity in various tumors, including intrahepatic cholangiocarcinoma [39], renal cell carcinoma [40], prostate carcinoma [41] and BCa [21].

Consistent with previous reports [4–6], our results revealed that the IHC-based Ki67 expression status was associated with BCa patients’ prognoses. Furthermore, we tried to explore the relationship between patients’ survival outcomes and the radiomics-predicted Ki67 expression status based on the SMOTE-LASSO model. Interestingly, BCa patients with radiomics-predicted high Ki67 expression had obviously poor DFS, indicating that the SMOTE-LASSO model not only had good performance in the prediction of Ki67 expression, but also may be a useful prognostic factor in BCa patients. In this way, the constructed radiomics signature provided a noninvasive, preoperative tool to predict Ki67 expression, and the radiomics-predicted Ki67 expression status was associated with prognosis in BCa. Traditionally, some preoperative clinical variables such as age, sex, tumor size and number of tumors have been found to be prognostic factors in BCa patients [42]. The inclusion of radiomics signature would provide additionally preoperative information for a better prediction of the prognoses and potentially help the treatment decision making in clinical practice. In addition, this new tool will potentially help physicians in developing a follow up post-operative plan for BCa patients.

There are some limitations in this study. First, it was a retrospective study and thus bias could not be avoided. Prospective study is warranted to further validate the radiomics signature. Second, single-center study cannot assess the generalizability of the radiomics signature in other centers; thus, a multicenter study is necessary to generalize the radiomics signature of this study.

In conclusion, we constructed a useful MRI-based radiomics signature for preoperatively predicting Ki67 expression in BCa with satisfactory diagnostic performance, which may have potential value for clinical decision-making in BCa.

## Supplementary Information

**Additional file 1.**

## Data Availability

The datasets used and/or analyzed during the current study are available from the corresponding author on reasonable request.

## Abbreviations

AUC: Area under the curve
BCa: Bladder cancer
DCA: Decision curve analyses
DCE: Dynamic contrast-enhancement
DFS: Disease-free survival
DICOM: Digital Imaging and Communications in Medicine images
ICC: The intra- and interclass correlation coefficients
IHC: Immunohistochemistry
LASSO: Least Absolute Shrinkage and Selection Operator
MRI: Magnetic resonance imaging
MIBC: Muscle-invasive bladder cancer
NMIBC: Non-muscle-invasive bladder cancer
NPV: Negative-predictive value
PPV: Positive-predictive value
ROC: Receiver operating curve
ROI: Region of interest
SMOTE: Synthetic minority over-sampling technique
SVM: Support vector machine
VI-RADS: Vesical Imaging-Reporting and Data System
VOI: Volumes of interest
T2WI: T2-weighted
