# Supplementary material for: Magnetic resonance imaging-based radiomics signature for preoperative prediction of Ki67 expression in bladder cancer
Source: Cancer Imaging. 2021 Dec 4;21:65. doi: 10.1186/s40644-021-00433-3 (PMC8642943; doi:10.1186/s40644-021-00433-3)
Supplement: Supplementary file 1 — Additional file 1. [file 40644_2021_433_MOESM1_ESM.doc]

**Table S1** R packages used in this study

| Statistical analysis | R package |
| --- | --- |
| mRMR | mRMRe |
| ROC | pROC |
| LASSO logistic regression | glmnet |
| SVM-RFE | e1071 |
| Calibration plot | rms |
| DCA curve | ggDCA |
